# Tilt Table Test: State of The Art

**Published:** 2003-10-01

**Authors:** Gonzalo Baron-Esquivias, Antoni Martinez-Rubio

**Affiliations:** * Cardiology Department, Hospital Universitario Virgen del Rocio. Seville; † Cardiology Department, Hospital Parc Tauli (Sabadell), Spain

**Keywords:** vasovagal syncope, tilt-table test

The loss of consciousness has been a subject of wonder and uncertainty in humans, and for this reason it has been the object of medical investigation since the beginning of time. Even actually, it is certainly an unresolved clinical problem. Many centuries ago, complicated exorcisms and remedies were used on these unfortunate patients, who upon regaining consciousness would find themselves soaked in miraculous liquids, ingesting curative potions, and often on the way to be burned accused of being possessed. In the seventeen century, physicians began to relate loss of consciousness and haemodinamic changes. William Harvey was perhaps the first to describe a circulatory response (vasovagal reaction) during a phlebotomy in the year 1628: “…Yet it fear or any other cause, or something do intervene through passion of the mind, so that the heart do beat more faintly, the blood will be no means pass through but drop after drop… ” [1]. During the nineteenth century, loss of consciousness was the object of studies and research, and the vagally mediated cardioinhibition, as a primary cause, was noted by Foster who proposed that profound bradycardia diminished cerebral perfusion to a level inadequate to maintain consciousness ” [2]. At this time, it was reported the first use of the tilt-table test ” [3]. Commonly referred to as fainting or loss of consciousness, from last century the preferred medical term is syncope, which itself is derived from the Greek term “syncoptein” meaning “to cut short”. Syncope is defined as the sudden loss of consciousness and postural tone with spontaneous recovery. In 1907, Gowers was the first person to use the term vasovagal syncope [4]. In 1918 was published the work in which Cotton and Lewis described for the first time the clinical characteristics that are still used today to recognize the syncopal reaction [5]. However, it was not till 1932 when Lewis described this reaction as being characterized by a combination of bradycardia, hypotension, and syncope, and he coined the term vasovagal syncope for the first time [6].

From the first studies with patients suffering loss of consciousness, it was observed that they all suffered a haemodynamic collapse which, though poorly understood initially, was analyzed with the diagnostic methods of the time period. In the past, those patients with recurrent syncopes were often subjected to a long, tedious, expensive, and all too often fruitless routine series of tests aimed at disclosing a potential aetiology. It was not until 1957 that one of the pioneering works on the use of the tilt-table test on such patients was published. In this work with university students, Weissler et al. tilted the individuals to 60° and observed 20% positive (abnormal) results. They induced syncope in others by oral administration of sodium nitrate 10-15 minutes before the test [7]. After studying patients with syncope of unknown aetiology and based on the known induction of a vasovagal response in young adults [8,9] as well as the results of aerospace studies [10-15], a group of English cardiologists conducted by Kenny and Sutton in 1986 published the first research paper on tilt testing [16]. In this Landmark study, they used the tilt-table test at an angle of 40° for 60 minutes on 15 patients with syncope of unknown aetiology, and on 10 control individuals. Nine of 15 patients (67%) and 1 control developed syncope during tilt.

From the time of this historic work to the present day, more than 830 papers have been published in which the keyword is *vasovagal syncope* and more than 975 with the term *tilt-table test*.

There is no question that this technique has helped for the understanding of the vasovagal syncope and its pathophysiology. However, many authors currently question this technique due to the many concerns that still need to be resolved:

1. Are clinical and induced vasovagal syncope equivalent?
2. Is a tilt-table test indicated in all patients with vasovagal syncope?
3. Is there a standardized protocol?
4. What is the result of a tilt-table test?
5. Is the tilt-table test useful for anything other than diagnostics?
6. Exist complications of the test?

## Correlation Between Clinical Syncope And Induced Syncope

In Medicine, many doubts exist regarding induced phenomena. Physicians should question the correlation between clinically spontaneous and induced results. Furthermore, is the clinical phenomenon reported by the patient equivalent to the induced? In the case of vasovagal syncope, it seems to be clear that they are equivalent. We can base this conclusion on three fundamental facts:

- Spontaneous syncope and induced syncope in the tilt-table test are associated with similar premonitory signs and symptoms. The most characteristic premonitory symptoms that are almost always described by patients are nausea, redness, perspiration, and abdominal discomfort. Clinical signs include marked pallor, bilateral mydriasis, and loss of postural tone [17-19].
- The temporal sequence of changes in blood pressure and heart rate during induced syncope parallel those seen with spontaneous syncope. Though earlier data from European studies placed doubt on this conclusion [20], it has been shown that in patients suffering from syncope, there was a demonstrable and reproducible neurovegetative imbalance [21,22]. This was manifested as an increase in central sympathetic stimulation preceding syncope [23] associated with a very acute decrease in cardiac output and a later drop in mean arterial pressure, as a result of the parasympathetic nervous activity and peripheral resistance [24] measured by means of microvascular Doppler with a decrease in subcutaneous blood flow [25].
- Plasma catecholamine levels measured before and after spontaneous and induced syncope are very similar.Above all, an increase in circulating catecholamines has been shown both in spontaneous syncope in the upright position [26-29] and in induced syncope with the tilt-table test [30], with maintained levels of norepinephrine and a five-fold increase in epinephrine levels [31], and with intervention of endogenous opioids and differences between experimental protocols [32].

## Who Should Undergo Tilt-Table Testing?

The ACC expert consensus document for tilt-table testing was published in 1996 with this summary of principals indications [17] divided into three general categories depending its warranty:

*Tilt table testing is warranted:*

1. If recurrent syncope or single syncopal episode in a high risk patient have occurred, whether or not the medical history is suggestive of neurally mediated (vasovagal) origin, and
  1.1: No evidence of structural cardiovascular disease.
  1.2: Structural cardiovascular disease is present, but other causes of syncope have been excluded by appropriate testing.
2. For further evaluation of patients in whom an apparent cause has been established (e.g. asystole, atrioventricular block), but in whom demonstration of susceptibility to neurally mediated syncope would affect treatment plans.
3. Parts of the evaluation of exercise-induced or exercise-associated syncope.

*Reasonable differences of opinion exist regarding utility of tilt table testing for:*

1. Differentiating convulsive syncope from seizures.
2. Evaluating patients (especially the elderly) with recurrent unexplained falls.
3. Assessing recurrent dizziness or presyncope.
4. Evaluating unexplained syncope in the setting of peripheral neuropathies or dysautonomies.
5. Follow-up evaluation to asses therapy of neurally mediated syncope.

*Tilt table test is not warranted after:*

1. Single syncopal episode, without injury and not in a high risk setting with clear-cut vasovagal clinical features.
2. Syncope in which an alternative specific cause has been established and in which additional demonstration of a neurally mediated susceptibility would not alter treatment plans.

*Potential emerging indications are:*

1. Recurrent idiopathic vertigo
2. Recurrent transient ischemic attacks
3. Chronic fatigue syndrome
4. Sudden Infant Death Syndrome (SIDS)

Later, and following the same kind of recommendations (class I, II and III), the European Society of Cardiology [33] published its guidelines in 2001. They recommended:

### Indications

#### Class I:

*Tilt testing is indicated for diagnostic purposes:*

1. In cases of unexplained single syncopal episodes in high risk settings (e.g. occurrence of, or potential risk for, physical injury or with occupational implications), or recurrent episodes in the absence of organic heart disease, or, in the presence of organic heart disease, after cardiac causes of syncope have been excluded.
2. When it will be of clinical value to demonstrate susceptibility to neurally-mediated syncope to the patient.

#### Class II:

*Tilt testing is indicated for diagnostic purposes:*

1. When an understanding of the haemodynamic pattern in syncope may alter the therapeutic approach.
2. For differentiating syncope with jerking movements from epilepsy.
3. For evaluating patients with recurrent unexplained falls.
4. For assessing pre-syncope or dizziness.

#### Class III:

1. For assessment of treatment.
2. A single episode without injury and not in a high risk setting.

### Diagnosis

#### Class I:

1. In patients without structural heart disease, tilt testing can be considered diagnostic, and no further test needs to be performed when spontaneous syncope is reproduced.
2. In patients with structural heart disease, arrhythmias or other cardiac causes should be excluded prior to considering positive tilt test results as evidence suggesting neurally mediated syncope.

#### Class II:

 The clinical meaning of abnormal responses other than induction of syncope is unclear.

## Is There A Standarised Protocol?

Since the first published work [16], numerous papers have been written on the methodology of this test that have increased our understanding of the pathophysiology of vasovagal syncope. Various protocols have been proposed in the literature and are explained below:

### Basal or Westminster Protocol:

In 1991, a group at the Westminster Hospital of London published their methodology showing the greater specificity of the test performed on a table with footboard support at an angle of 60° for 45 minutes without intravenous cannulation or infusion of vasoactive substances. They described a sensitivity of 75% and a specificity of 93% [34]. Doubts exist about the duration that should be used during tilt testing. To address this question, Stein et al analyzed data from 11 published studies using this protocol and performed tilt testing on 213 patients for 30 to 60 minutes. They demonstrated that diagnostic accuracy is not greatly influenced by duration of the test after 30 minutes, varying from 60% to 84% [35]. Furthermore, this protocol has been used less and less, in spite of what was initially described, since the true sensitivity appears to be lower. In a study of more than 1,000 consecutive patients, our group found a sensitivity of 24.4% but with a significant decrease with age. Thus, the rate of positive responses in young subjects (<20 years old) was 41% but it decreased to only 19% in subjects older than 60 years (p<0.05) [36].

### Isoprenaline Protocol:

Mainly proposed by North American authors, this method consists of an initial phase of tilting without drug administration and a later phase in which isoprenaline is administered by intravenous infusion at different rates, using an angle of 70-80°. Between the various suggested protocols using this drug, one of the most commonly used is the published by Almquist [37]. In this, following a basal phase of 10 minutes of tilting at 80°, progressive doses from 1 to 5 mg/min of isoprenaline are infused. This method results in a sensitivity of 87% and a specificity of 85%. Other authors have criticized this protocol reporting lower sensitivity (75%) and lack of specificity [38]. This, in addition to secondary effects later described, led to the use of lower doses. Morillo et al [39] advocate this, and achieved a sensitivity of 61% and a specificity of 93%. Other authors have compared high and low doses of this substance and achieved better results with low doses (2 mg/min), with a sensitivity of 64% and specificity of 88.9% [40]. With the intention to reduce the long necessary time for this technique, a protocol using this substance was recently published in which the basal phase is eliminated, using only the isoprenaline phase [41].

### Edrophonium Protocol:

After a 45-minute basal phase, a 10 mg intravenous bolus of this substance has been used and tilting is prolonged for another 20 minutes [42]. These authors found a high rate of positive responses at two different angles (60° and 80°). Since this study used a femoral arterial catheter, the results may be questioned and certainly no other group has reported experience with this protocol.

### Clomipramine Protocol:

A group in Greece recently reported the use of this protocol without a basal phase, infusing 5 mg of this substance intravenously, and holding the table for 20 minutes [43,44]. This protocol resulted in a sensitivity of 80% and a specificity of 95.4%.

### Nitroglycerine Protocol (Italian Protocol):

In 1994 Raviele et al. studied in 40 patients with syncope and 25 controls with previous negative tilt-table test results, a new protocol at 80° using intravenous nitroglycerine. They reported a sensitivity of 53% and a specificity of 92% [45]. Several years later, this author used a 60° angle for 45 minutes followed by sublingual administration of 300 mg. nitroglycerine followed by 20 minutes of further tilt testing to analyze the response of 235 patients and 35 controls. This method yielded a sensitivity of 51% and a specificity of 94% [46]. Later works were published [47-51] until the definitive publication of the Italian protocol [52], which recommends 20 minutes of tilting at 60° without drugs and without cannulation of peripheral veins. This was followed by an infusion of 400 mg of nitroglycerine sprayed sublingually, followed by 15 minutes of tilting. This protocol yielded a sensitivity of 62% and a specificity of 92%. We recently reported our experience with this protocol based on data of 426 patients (sensitivity of 67.5%; specificity of 85.7%) [53].

In view of all these data, we may conclude that there is not a standardized protocol for tilt test. However, for selection of a tilt test protocol, one should take into consideration that while the protocol without drugs (Westminster) has shown the highest specificity, the low sensitivity of this method suggests as necessary to use a drug induction phase. North American authors have preferred to use isoprenaline while most Europeans use nitroglycerine. Both strategies are valid since when both protocols are compared (randomly) similar results are achieved [54,55].

### General recommendations independent of protocol:

**Figure 1:**
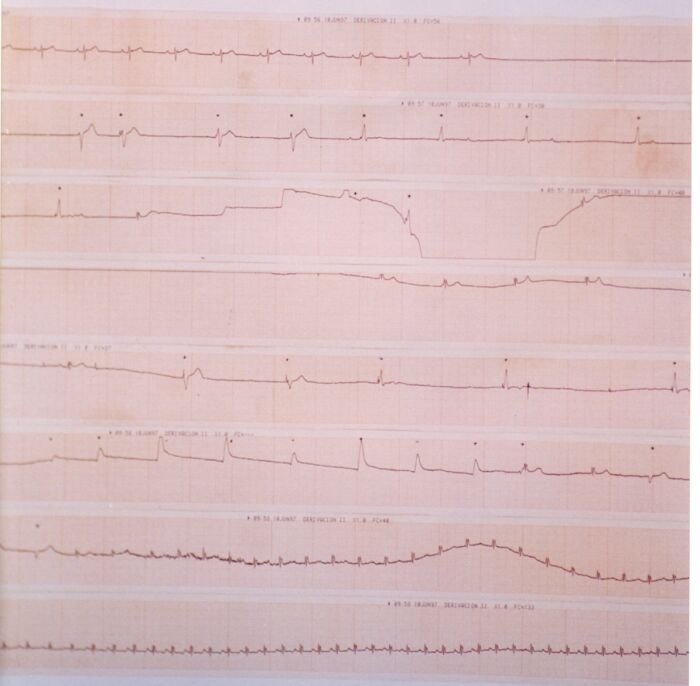
Continuous ECG trace of syncope development during HUT. **Top:** Baseline ECG before Tilting. ** Bottom:** Continuous ECG beginning with bradycardic sinus rhythm followed by asystole and occasionally occurring escape rhythm and beats (*) induced by vigorous blows to the precordium. The bradycardic arrhythmic events reverted to sinus tachycardia during Trendelemburg position and after atropine.

The test should be performed in a quiet room with the lights slightly dimmed. Although the broad majority of groups report to perform the test during the morning, obviously, it may be undertaken during afternoon or at the evening. However, circadian patterns might influence the results of the test. It is important that the patient has fasted at least 2 hours before the test. In order to reduce the probability of a vasovagal response to venous cannulation, it has been proposed that patients remain in a supine position for 20 to 45 minutes before the test. Based on specificity reduction observed with cannulation [56], some protocols avoid its use and thus, supine position should be reduced to 5 minutes in those cases. Arterial blood pressure should be measured continuously. It is, therefore, recommended to use beat-to-beat non-invasive blood pressure measurement systems. Intra-arterial cannulation has sometimes been used, but this can modify the specificity of the test in children and the elderly [33]. Intermittent measurements with a sphygmomanometer have been widely used. Although it is not the most desirable method, the price of continuous measurement devices makes it very commonly used in clinical practice. When performing the test, it should be possible to tilt the patient to the desired angle smoothly and rapidly, and it should also be possible to return quickly to a supine position (<10 sec.) when the test is terminated to avoid the consequences of a very prolonged loss of consciousness [33]. Our group has shown that the duration of time for changing to Trendelenburg position can have a negative influence on the duration of asystoles observed in some patients during the test (Figure 1). For this reason, we recommend that the necessary time from head-up tilt to Trendelenburg should be as short as possible [57]. Tilt tables should be equipped with footboard supports. An experienced nurse or technician should be continuously present during the test. The need to have a physician continuously present during the test has not been well established. However, since the risk for the patient during the test is very low, it seams to us as sufficient that physicians should be nearby and immediately available if a problem arises.

## What Are The Results Of A Tilt-Table Test?

When a patient undergoes a tilt-table test, two main responses may be observed. The first is a normal or negative response, which is defined as slight fluctuations in systolic and diastolic blood pressure and in heart rate, without any other abnormalities. The other response is the abnormal or positive response, which is characterized by a loss of consciousness following various haemodynamic patterns. Although vasovagal syncope is the diagnostic objective of the test, from the first studies it has been observed that there are others different positive responses (Table 1) [58].

**Figure 2:**
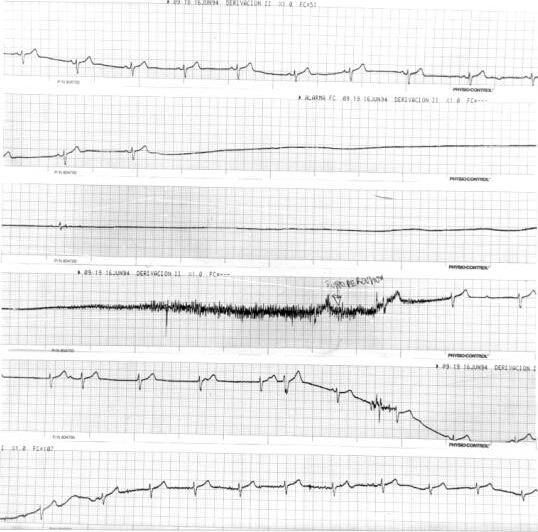
Continuous ECG trace of syncope development during HUT. After symptom initiation, 15 sec. of relative bradycardia is abruptly followed by a 31 sec. asystole period. After tilt toward Trendelenburg position, patient presented tonic-clonic movements and muscular jerks

Based on changes in arterial blood pressure and heart rate preceding the vasovagal reaction, different patterns have been recognized. Overall, two of these are seen most commonly [33]. The typical or classic pattern is characterized by a rapid and complete initial reflex phase of adaptation to the upright position with a stabilization of blood pressure and heart rate (which suggests normal baroreflex function) that lasts until the rapid onset of the vasovagal reaction (Figure 2). Patients exhibiting this kind of pattern tend to be young and healthy. They have a history of multiple syncopal episodes over many years. In many cases the first syncopal episode occurred at the age of 10 to 15 years. Secondary trauma is uncommon in those patients. This pattern represents a hypersensitive autonomic nervous system that responds excessively to various stimuli. On the other hand, a different pattern is often observed which is characterized by an inability to achieve adaptation to the upright position. This results in a progressive drop in arterial blood pressure and heart rate, which occurs until symptoms appear. This is the so-called hypotensive pattern, in which the cause of symptoms appears to be the inability to adapt to external influences (hyposensitive autonomic nervous system function). Patients who show this kind of response tend to be elderly and have (many) associated diseases. They have a short history of syncope with few episodes per patient. The syncopal episodes begin later in life, suggesting that they occur as a result of some underlying progressive dysfunction. This pattern is similar to the pattern observed in patients with autonomic nervous system dysfunction and suggests the existence of a superimposition of the typical vasovagal syncope and other more complex pathological conditions of the autonomic nervous system. The tilt-table test can be useful in differentiating between these two syndromes.

The typical or classic positive response has been initially classified as vasodepressor, cardioinhibitory, and mixed. The occasional appearance of asystoles during this typical response prompted to Sutton et al in 1992 to use the details of the haemodynamic response to the test to propose a classification scheme which has been recently modified and it has become an easy and useful tool [59,60] (Table 2).

The decision of when to terminate the test will influence the type of response that is observed. For this reason, it is recommended that in order to ensure correct classification of the response, the test should be terminated at the same moment that consciousness and postural tone are lost (when syncope occurs). A premature interruption will result in underestimation and a delayed termination will result in an overestimation of the cardioinhibitory response and will expose the patient to the consequences of a prolonged loss of consciousness. However, there has not been a consensus on this subject, and many physicians feel that it is sufficient to terminate the test when a sustained drop in blood pressure is observed simultaneously with symptoms.

## Is The Tilt Test Useful For Anything Other Than Diagnostics?

Evaluation of the response to treatment: Many authors have attempted to show the benefits of the various proposed treatments for vasovagal syncope evaluating their ability to produce a negative result on a patient who had previously presented a positive response to the test (ACC Class II recommendation in 1996 [17]. However, this has been criticized [33,61,62]. In order to use the test for evaluation of therapeutic options, two conditions need to be fulfilled: the test and a positive response to the test must be highly reproducible and must have high predictive value of later outcome. Reproducibility of the tilt-table test has been widely studied. Overall, reproducibility of an initial negative response (85%-94%) is greater than it is for a positive response (31%-92%). Furthermore, data from controlled studies show that approximately 50% of patients with a positive basal test result will present a negative test if it is repeated under treatment or with a placebo [63]. Additionally, short-term studies are not predictive of the long-term outcome under therapy (such as definitive stimulation). Thus, actual data show that the use of this test to evaluate the effectiveness of different treatments is subject to significant limitations [33].

### Usefulness as therapy:

Based on the above mentioned tendency of response negativization after repeated test, and due to the absence of any fully effective treatment for vasovagal syncope, various authors have proposed the use of the tilt-table test as a form of therapy for vasovagal syncope, the so-called “tilt training” [64,65]. These authors propose the use of the upright position supported against the wall for short and repeated time periods on successive days to train the body to withstand the upright position, thus significantly reducing the recurrence of syncope.

## Complications

The tilt-table test is a safe procedure, and the occurrence of complications is very low. Although extremely prolonged asystolic pauses have been reported, the occurrence of these asystoles during a positive response cannot be considered a complication since this is an end-point of the test [57,60]. A rapid return to a supine position (or better to a Trendelemburg position), as soon as syncope occurs, is usually enough to prevent or limit the consequences of a prolonged loss of consciousness, however brief resuscitation maneuvers are occasionally required [66]. Complications have not been reported in relation to the Westminster protocol or with the use of nitroglycerine, but minor side effects occur commonly and include palpitations with the use of isoprenaline and headaches with nitroglycerine. However, using isoprenaline protocols, high doses of the drug are well tolerated in only 62% of patients, must be reduced in 33% and discontinued in 4.2%. Side effects include tachycardia (45%), nausea (35%), chest pain (2.2%), arrhythmias (6%), and other effects (10.3%) [67]. Vasospasm has been described several times with the use of this substance [68]. Life-threatening ventricular arrhythmias have been described with isoprenaline in the presence of ischemic heart disease or sinus dysfunction. Atrial fibrillation can be induced during or after a positive tilt test and is usually self limiting.

## Conclusions

Vasovagal syncope is the most frequent form of syncope of unknown aetiology. Since 1986, the tilt-table test has become an important tool in the diagnosis of vasovagal syncope. Before this test was introduced, several patients were submitted to a multitude of tests although most of them did little more than waste of resources. The similarity between clinical and induced vasovagal syncope during the test shows its value. The tilt-table test yields a range of positive results from 60% to 70%, with a specificity greater than 85%. The results are also highly reproducible (data similar to provocation diagnostic tests such as the treadmill test) when drug-based protocols are used. This, with the absence of serious complications and very small number of side effects, makes probably the nitroglycerine protocol as the actually most recommended test for clinical practice.

## Figures and Tables

**Table 1:**
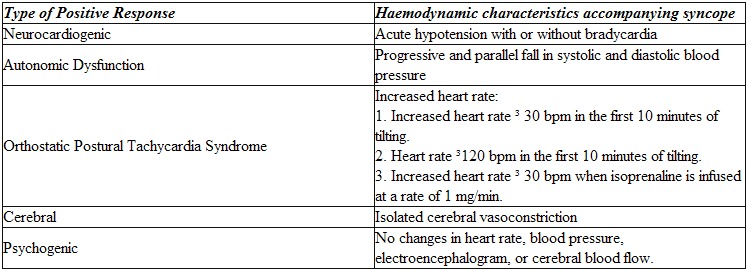
Type of positive response during the tilt-table test

**Table 2:**
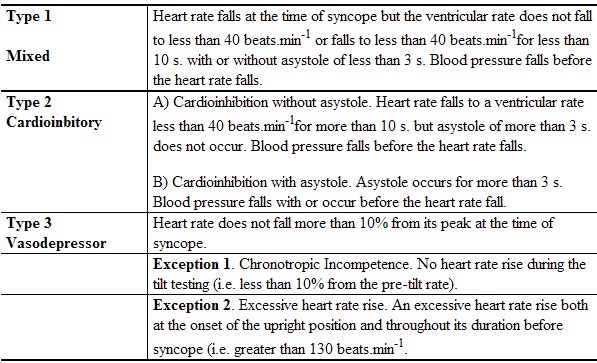
Classification of positive responses to tilt testing
